# Feasibility of non‐anesthesiologist‐administered sedation with dexmedetomidine and midazolam during endoscopic submucosal dissection of upper gastrointestinal tumors

**DOI:** 10.1002/deo2.70045

**Published:** 2024-12-21

**Authors:** Kenji Ishido, Satoshi Tanabe, Gen Kitahara, Yasuaki Furue, Takuya Wada, Akinori Watanabe, Hiromi Matsuda, Hirotsugu Okamoto, Chika Kusano

**Affiliations:** ^1^ Department of Gastroenterology Kitasato University School of Medicine Kanagawa Japan; ^2^ Department of Anesthesiology Kitasato University School of Medicine Kanagawa Japan; ^3^ Department of Gastroenterology Ebina General Hospital Kanagawa Japan

**Keywords:** dexmedetomidine, endoscopic submucosal dissection, non‐anesthesiologist, on‐the‐job training, sedation

## Abstract

**Objectives:**

The efficacy and safety of a sedation regimen combining dexmedetomidine and midazolam during endoscopic submucosal dissection for upper gastrointestinal tumors remains unclear. In this study, we aimed to evaluate the efficacy and safety of this sedation regimen, where non‐anesthesiologists performed sedation.

**Methods:**

Sixty‐eight patients who underwent endoscopic submucosal dissection for upper gastrointestinal tumors, sedated by non‐anesthesiologists, were retrospectively evaluated. The sedation was performed by non‐anesthesiologists as part of on‐the‐job training (OJT) under anesthesiologists' supervision. Each non‐anesthesiologist received OJT at least thrice. Proficiency levels were assessed during the third OJT session. The target sedation depth was a Richmond Agitation‐Sedation Scale of −2 to −4, with 2 L/min of oxygen delivered via a nasal cannula at sedation initiation. The treatment completion rates, which measured efficacy and safety, were assessed by the frequencies of respiratory depression, hypotension, and bradycardia.

**Results:**

The study included 14, 52, and two patients with superficial esophageal cancer, early gastric cancer, and gastric adenoma, respectively. The median treatment time was 68 and 84 min for superficial esophageal cancer, early gastric cancer, and adenoma, respectively. Endoscopic submucosal dissection was completed in all patients. No severe sedation‐related adverse events were reported; however, peripheral arterial oxygen saturation <90%, hypotension, and bradycardia occurred in 1 (1.5%), 30 (44.1%), and 30 patients (44.1%), respectively. All 22 non‐anesthesiologists who underwent the proficiency evaluation passed the test.

**Conclusions:**

A sedation regimen combining dexmedetomidine and midazolam can be feasibly administered by non‐anesthesiologists. Further studies are needed to verify the effectiveness of OJT.

## INTRODUCTION

The role of sedation in endoscopic procedures is to alleviate patient anxiety and distress, thereby improving patient acceptance and satisfaction.^1^ Sedation during these endoscopic procedures can cause airway compression by the endoscope, necessitating close monitoring of respiration. Data from the American Society of Anesthesiologists Closed Claims database suggest that sedation outside the operating room due to oversedation and inadequate oxygenation or ventilation during monitored anesthesia care poses significant risks.^2^

In Japan, there is a shortage of anesthesiologists, making it difficult for them to oversee all sedation procedures.^3^ Consequently, non‐anesthesiologists, particularly gastroenterologists, often manage the sedation of patients despite having little training in this area. These patients frequently require prolonged treatment or have higher sedation related‐risks (Table 1). Therefore, a system to support safe sedation is required to ensure patient safety.^1^, ^4^

**TABLE 1:** Reasons for sedation problems when administered by non‐anesthesiologists (gastroenterologists).

The issue of the need for sedation	Improved diagnosis of early‐stage cancer has increased the need for sedation during endoscopy and treatment. Diversification of sedation agents.
A matter of recognition	Lack of awareness that sedation improves the quality of examination and treatment but may compromise patient safety. Difference between sedation and general anesthesia.
Training system issues	Non‐anesthesiologists (particularly gastroenterologists) often receive little training in sedation.
Anesthesiologist issues	Lack of manpower of anesthesiologists in Japan.

At our hospital, midazolam (MDZ) or propofol is commonly used for sedation during endoscopic procedures. However, propofol has a narrow therapeutic range and carries risks of respiratory depression and circulatory instability.^5^ Recently, dexmedetomidine (DEX, Maruishi Pharmaceutical Co., Ltd.) has been approved for use under insurance coverage for “sedation during non‐intubated surgery and procedures under local anesthesia,” and is now widely used as a sedative during endoscopic procedures.

Sedation using a combination of DEX and MDZ for endoscopic retrograde cholangiopancreatography is reportedly feasible in adults aged >80 years when administered by non‐anesthesiologists.^6^ However, the use of DEX for sedation in our hospital during endoscopic procedures was restricted due to safety concerns related to cardiac dynamics.

To safely implement a sedation regimen combining DEX and MDZ for endoscopic submucosal dissection (ESD) of upper gastrointestinal tumors, we introduced a sedation licensing system for non‐anesthesiologists. This program consists of a web‐based training course, on‐the‐job training (OJT) for a minimum of three sessions per physician under anesthesiologist supervision, and a proficiency evaluation during the third OJT session. Only non‐anesthesiologists who pass the sedation licensing system were allowed to use the combined DEX and MDZ sedation regimen in practice.

We retrospectively evaluated the efficacy and safety of a sedation regimen administered by non‐anesthesiologists in patients undergoing ESD for upper gastrointestinal tumors.

## METHODS

### Patients

We retrospectively examined the efficacy and safety of a sedation regimen in consecutive ESD cases for upper gastrointestinal tumors. Sedation in these cases was administered by a non‐anesthesiologist under OJT using the sedation regimen of DEX and MDZ in the Department of Gastroenterology at Kitasato University School of Medicine, between January 2021 and August 2023. The OJT was conducted under the supervision of an anesthesiologist, with sedation performed by personnel separate from the endoscopist conducting the procedure. Cases without complete proficiency assessments for the first, second, and third OJT sessions were excluded.

The clinicopathological characteristics of the patients included age, sex, body mass index (BMI), Eastern Cooperative Oncology Group performance status, American Society of Anesthesiologists physical status classification (ASA‐PS),^7^ Charlson Comorbidity Index,^8^ presence of cardiovascular or respiratory disease, use of antithrombotic medication, smoking history (categorized as never/light [<30 pack‐years] or heavy [≥30 pack‐years]), alcohol consumption (categorized as never, current drinker, or existing drinker) and pure ethanol equivalent, lesion site (upper, middle, or lower thoracic esophagus, and upper, middle, lower, or remnant stomach), macroscopic type (protruded or flat and depressed), presence of scarring, histological type (squamous cell carcinoma, adenocarcinoma, or other in esophagus; differentiated or undifferentiated in the stomach), diameter of the resected specimen (mm), diameter of the resected tumor (mm), depth of invasion, complete en bloc resection, curability criteria for endoscopic resection in the stomach,^9^ and treatment duration. Treatment duration was defined as the time from insertion to removal of the endoscope. Adverse events were evaluated using Common Terminology Criteria for Adverse Events Version 5.0.^10^ Delayed bleeding was defined as “hematemesis or hemorrhage” or “Hb <2 g/dL,” with endoscopically confirmed bleeding within 28 days post‐ESD. Perforation was defined by the absence of muscularis propria and the presence of intra‐abdominal free air or mediastinal emphysema.

Procedure availability was assessed based on the occurrence of body movements during upper ESD that interrupted treatment. Safety was evaluated by the frequency of respiratory depression (percutaneous peripheral arterial oxygen saturation [SpO_2_] <90% or the need for nasal airway insertion), hypotension (systolic blood pressure <90 mmHg), and bradycardia (pulse <50 bpm).

Informed consent, with an opt‐out option, was obtained from all patients in compliance with the Japanese guidelines for human medical research. This study adhered to the principles outlined in the Declaration of Helsinki and was approved by the Ethics Committee of the Kitasato University School of Medicine Hospital (B21‐207).

### Sedation licensing system

**FIGURE 1:**
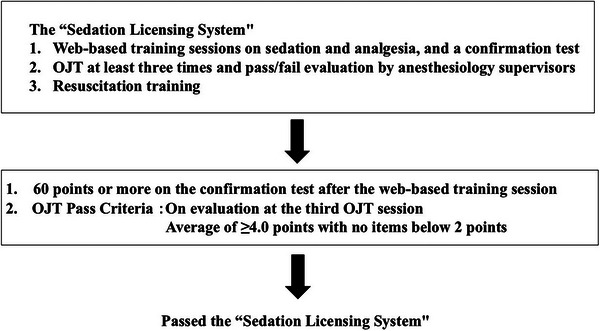
Sedation Licensing System

OJT for non‐anesthesiologists is part of the sedation licensing system, which consists of the following steps (Figure 1):

1) Non‐anesthesiologists attended a web‐based training course on sedation and analgesia provided by anesthesiologists. The course covered the purpose of sedation, target sedation depth, pharmacologic actions of sedatives and analgesics, mechanism of action, contraindications, administration methods, antagonists, and response to emergencies. After the training session, a confirmatory test was administered, and a score of ≥60 out of 100 points was considered acceptable.

2) Non‐anesthesiologists who passed the web‐based training session were administered OJT at least three times per physician using the sedation regimen prepared by the anesthesiologist.

Initially, the anesthesiologist provided detailed instructions according to the “Flowchart for sedation regimen,” such as adjustments to DEX dosage or additional doses of buprenorphine hydrochloride (BPN; Table 2). In the second OJT session, a non‐anesthesiologist primarily managed sedation, with guidance provided as needed. By the third session, non‐anesthesiologists almost exclusively managed sedation and were evaluated by the anesthesiologist on a 5‐point scale across 11 items (Table 3). Passing was determined by achieving a mean score of ≥4.0, with no individual score <2 in each category.

**TABLE 2:** Flowchart for the sedation regimen with dexmedetomidine and midazolam.

Routine work	DEX loading	≥80 years	DEX loading at 6 µg/kg/h for 5 min, continuous administration of DEX at 0.4 µg/kg/h
		<80 years	DEX loading at 6 µg/kg/h for 10 min, continuous administration of DEX at 0.4 µg/kg/h
	DEX maintenance dose		DEX 0.4 µg/kg/h of continuous administration
	(Initial dose) Midazolam and buprenorphine hydrochloride	≥60 years	Midazolam 1 mg i.v. and buprenorphine hydrochloride 0.1 mg i.v.
		<60 years	Midazolam 2 mg i.v. and buprenorphine hydrochloride 0.2 mg i.v.
	When inserting an overtube		Additional doses of buprenorphine hydrochloride 0.1 mg i.v.
	More than 90 min after the first dose		Additional doses of buprenorphine hydrochloride 0.1 mg i.v.
Improvised work	During body movement	≥60 years	Midazolam 1 mg i.v. and DEX increased each by 0.1 µg/kg/h (up to 0.7 µg/kg/h)
		<60 years	Midazolam 2 mg iv and DEX increased each by 0.1 µg/kg/h (up to 0.7 µg/kg/h)
	During severe body movements that are uncontrollable by one person		Additional doses of buprenorphine hydrochloride 0.1 mg i.v.; administration 5 min apart, up to two consecutive doses
	The total dose of midazolam exceeds 5 mg, and the upper limit of DEX (0.7 ug/kg/h)		Additional doses of buprenorphine hydrochloride 0.1 mg i.v. Can be administered as many times as needed, 20 min apart
	Adjustment of DEX	Tachycardia (e.g., >80 beats per min [bpm]) and sufficient blood pressure (e.g., 140 mmHg)	DEX increased each by 0.1 µg/kg/h (up to 0.7 µg/kg/h)
		Bradycardia (e.g., <40 bpm sustained and blood pressure <100 mmHg)	DEX decreased each by 0.1 µg/kg/h
	Heart rate in the range of 40 bpm		DEX decreased each by 0.1 µg/kg/h, or administration of ephedrine hydrochloride 4 mg i.v. or atropine sulfate 0.5 mg i.v.
	Blood pressure <80 mmHg		Administer ephedrine hydrochloride 4 mg i.v. It can be administered as many times as needed, 5 min apart.
	Airway obstruction		Perform airway clearance, such as mandibular elevation and insertion of the nasal airway tube. If there is no improvement, decrease dexmedetomidine.
	Respiratory arrest		DEX is stopped, and the breathing is supported until the call response is made.

Abbreviations: DEX, dexmedetomidine; i.v., intravenous injection.

**TABLE 3:** Evaluation items of on‐the‐job training.

Evaluation details	
1	Loading
2	Age‐appropriate drug adjustment
3	Coping with body movement
4	Adjustment of DEX maintenance rate
5	Additional dose of buprenorphine hydrochloride
6	Airway management
7	Circulation management
8	Vital sign check
9	Check the sedation level
10	Timing of return to the room
11	Instructions for returning to the room

*Notes*: “The evaluation contents are rated on a 5‐point scale.”

1: All are poorly understood; 2: Partially understood but not fully; 3: I can understand and act if pointed out; 4: I can make decisions on my own initiative with my own criteria; 5: Accurate knowledge and the ability to act safely.

OJT pass criteria: On evaluation after the third session, an average of ≥4.0 points with no items <2 points.

To obtain sedation licensure, participants were required to complete web‐based training, OJT, and resuscitation training. An anesthesiologist assessed whether a non‐anesthesiologist could safely administer sedation independently. Following this evaluation, the anesthesiologist reviewed the OJT scores and provided feedback.

The third OJT session required a mandatory evaluation by the anesthesiologist, whereas evaluation for the first and second sessions was optional.

### Sedation regimen

The sedation regimen was developed by an anesthesiologist who visited the endoscopy center and adapted it to the actual conditions of the endoscopic procedures.

DEX was initiated at a continuous infusion rate of 6 µg/kg/h as a loading dose. The loading time for DEX was 10 min for patients aged <80 years and 5 min for those aged ≥80 years. Subsequently, the DEX infusion was reduced to a maintenance dose of 0.4 µg/kg/h. For patients aged <60 years, MDZ 2 mg and BPN 0.2 mg were administered, while those aged ≥60 years received a bolus of MDZ 1 mg and BPN 0.1 mg (Table 2). The target sedation depth was defined as a Richmond agitation‐sedation scale (RASS)^11^ of −2 to −4. During sedation, patients exhibiting movement, sustained blood pressure below 80 mmHg, persistent bradycardia of approximately 40 bpm, airway obstruction, or respiratory arrest were promptly managed appropriately (Table 2).

### Monitoring of patients

During ESD, the blood pressure, pulse, SpO_2_, electrocardiogram, and respiratory rate were monitored. Blood pressure was recorded every 5 min. Oxygen was administered at 2 L/min via a nasal cannula at the initiation of sedation and increased if SpO_2_ dropped to ≤93%.

### Exit criteria

After endoscopic treatment, DEX was discontinued, and flumazenil, an MDZ antagonist, was administered. The following criteria had to be met before the patient was allowed to leave the recovery area: 1) patient could respond to their name, 2) SpO_2_ ≥94% (nasal cannula with oxygen flow at 3 L/min was permissible), 3) no seesaw breathing and a respiratory rate between 10 and 25 breaths/min, 4) systolic blood pressure within 80–170 mmHg and pulse rate of 50–110 beats/min, and 5) no safety concerns.

### Statistical analysis

All statistical analyses were performed using SPSS, version 27 (SPSS Japan Inc.). The results of the OJT scoring by anesthesiologists were statistically analyzed using the Mann–Whitney *U* test for each evaluation item. All *p*‐values were two‐sided, and a *p*‐value <0.05 was considered statistically significant.

## RESULTS

**FIGURE 2:**
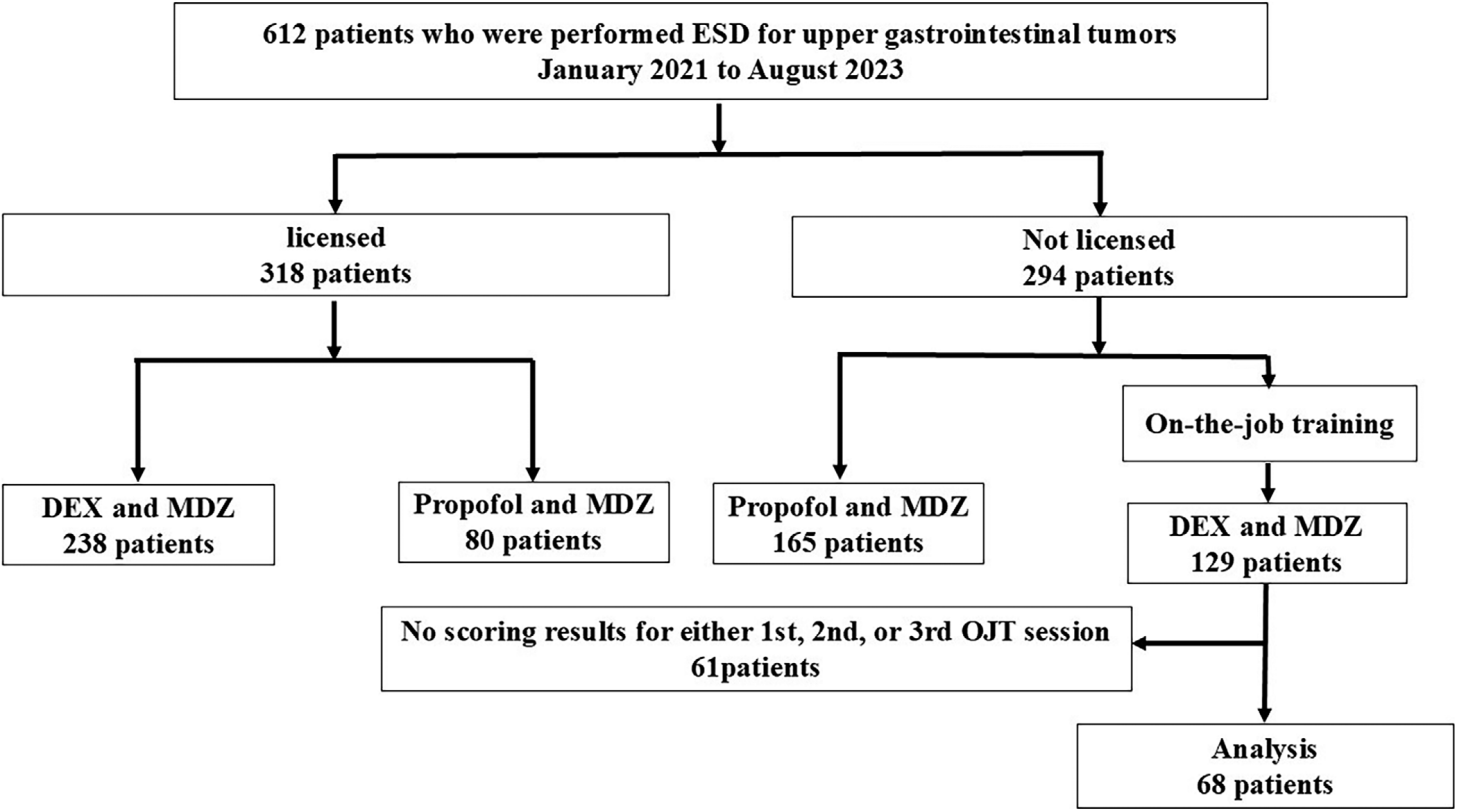
Flowchart 318 patients received sedation administered by licensed non‐anesthesiologists. Sedation with DEX and MDZ was used in 238 patients. 294 patients received sedation administered by non‐licensed non‐anesthesiologists. 129 patients were sedated with DEX and MDZ according to the sedation regimen with OJT. Sixty‐eight patients with first, second, and third OJT scoring results were included in the analysis. ESD, endoscopic submucosal dissection; DEX, dexmedetomidine; MDZ, midazolam; OJT, on‐the‐job training.

ESD procedures for upper gastrointestinal tumors were performed on 612 patients between January 2021 and August 2023. Among these, 318 patients received sedation administered by licensed non‐anesthesiologists. Sedation with DEX and MDZ was used in 238 patients, while propofol and MDZ were used in 80 patients. On the other hand, 294 patients received sedation administered by non‐licensed non‐anesthesiologists. Sedation with propofol and MDZ was used in 165 patients, 129 patients were sedated with DEX and MDZ according to sedation regimen with OJT. Sixty‐eight patients with first, second, and third OJT scoring results were included in the analysis (Figure 2). These cases included 14, 52, and two lesions of superficial esophageal cancer, early gastric cancer, and gastric adenoma, respectively. Of the 24 physicians who performed the ESD procedures, eight were gastrointestinal endoscopists, whereas 16 were non‐specialists.

The median age of the patients was 74 (range, 53–91) years for superficial esophageal cancer and 75 (range, 35–86) years for early gastric cancer and gastric adenoma (Table 4). The median BMI was 23.0 kg/m^2^ in patients with superficial esophageal cancer and 23.3 kg/m^2^ in those with early gastric cancer and gastric adenoma. None of the patients had a BMI >35 kg/m^2^. No patients with COPD were treated with home oxygen therapy. All ESD procedures for superficial esophageal cancer, early gastric cancer, and gastric adenoma were completed. The en‐bloc resection rate was 92.2% (13/14) for superficial esophageal cancer and 100% (54/54) for early gastric cancer and gastric adenoma. The median treatment duration was 68 (range, 40–142) min for superficial esophageal cancer and 84 (range, 24–208) min for early gastric cancer and gastric adenoma.

**TABLE 4:** Clinicopathological characteristics.

		Superficial esophageal cancer (*n* = 14)	Early gastric cancer and gastric adenoma (*n* = 54)
Age	Median (range; years)	74 (53–91)	75 (35–86)
Sex	Male/female	13/1	43/11
Body Mass Index	Median (range; kg/m^2^)	23.0 (18.9–27.4)	23.3 (16.4–34.4)
ECOG Performance Status	0/1	13/1	42/12
ASA physical status	I, II/III	12/2	39/15
Charlson comorbidity index	0,1/≥2	8/6	37/17
CPAP use for sleep apnea syndrome	Yes/no	2/12	3/51
Cardio‐cerebral vascular disease or respiratory disease	Positive/negative	2/12	11/43
Antithrombotic drugs	Yes/no	5/9	18/36
Smoking, pack‐years	Never/light, <30 and heavy, ≥30	1/5/8	23/13/18
Alcoholic intake	Never/current/habitual drinker	1/11/2	17/33/4
Pure ethanol equivalent (current or habitual)	≥60 g/<60 g	2/12	1/36
Location	Ut/Mt/Lt/Ae (Esophagus)	0/11/3	−
	U/M/L/remnant (Stomach)	−	13/17/21/2
Macroscopic type	Protruded/flat and depressed	4/10	22/32
Scar	Positive/negative	0/14	1/53
Histopathological type	SCC/adenocarcinoma (esophagus)	11/3	−
	Differentiated/un‐differentiated /adenoma/others*(stomach)	−	47/3/2/2
Diameter of resected specimen Length	Median (range; mm)	28 (18–45)	36 (15–70)
Diameter of resected tumor length	Median (range; mm)	11 (2–35)	13 (3‐45)
Depth of invasion	EP/LPM or SMM/MM/SM1/SM2	6/6/1/1/0	−
	M/SM1/SM2	−	45/4/3
Complete en block resection	% (*n*)	92.9 (13/14)	100 (54/54)
Criteria for curability of endoscopic resection (stomach)	eCura A/eCura B/eCura C‐2	−	44/2/5
Treatment duration	Median (range; min)	68 (40–142)	84 (24–208)

*Note*: CPAP, continuous positive airway pressure; ECOG, Eastern Cooperative Oncology Group; others*, neuroendocrine tumor G1

The median total dose of DEX was 100 (range 52–260) µg. The median total dose of MDZ was 2 (range, 1–7) mg, with additional MDZ administered to 53 patients (77.9%). The median total dose of BPN was 0.2 (range, 0.1–0.3) mg, and additional BPN was administered to 42 patients (61.8%; Table 5).

**TABLE 5:** Total infusion dose of dexmedetomidine, midazolam, and buprenorphine hydrochloride.

		*n* = 68	Superficial esophageal cancer (*n* = 14)	Early gastric cancer and gastric adenoma (*n* = 54)	*p*‐value
Total dose of DEX	Median (range) µg	100 (52–260)	100 (60–168)	96 (52–260)	0.933
Total dose of MDZ	Median (range), mg	2 (1–7)	2 (1–6)	2 (1–7)	0.821
Additional dose of MDZ	Yes/no	53 (77.9%)/15 (22.1%)	10 (71.4%)/4 (28.6%)	43(79.6%)/11(21.4%)	0.513
	Median (range), mg	1.5 (1–6)	1.5 (1–4)	1 (1–6)	0.906
Total dose of BPN	Median (range), mg	0.2 (0.1–0.3)	0.2 (0.1–0.3)	0.2 (0.1–0.3)	0.314
Additional dose of BPN	Yes/no	42 (61.8%)/26 (38.2%)	6 (42.9%)/8 (57.1%)	36(66.7%)/18 (33.3%)	0.105
	Median (range), mg	0.1 (0.1–0.2)	0.1	0.1 (0.1–0.2)	0.078

Abbreviations: BPN, buprenorphine; DEX, dexmedetomidine; MDZ, midazolam

One case of superficial esophageal cancer experienced an intraoperative perforation (7.1%). Among cases of early gastric cancer and gastric adenoma, nine adverse events (16.7%) occurred, including five cases (9.3%) of delayed bleeding, one case (1.9%) of intraoperative perforation, one case (1.9%) of delayed perforation, and two cases (3.7%) of aspiration pneumonia. None of the patients experienced serious adverse events, and all were managed conservatively.

SpO_2_ levels <90% were observed in one patient (1.5%), but oxygenation was improved with supplemental oxygenation. Airway stenosis occurred in one patient (1.5%), which improved after the endoscope was removed without the need for a nasal airway tube, though the procedure was interrupted. This patient had an ASA‐PS score of 3, a Charlson Comorbidity Index score of 4, a history of renal dysfunction, and a history of heavy alcohol drinking, which may have contributed to oversedation.

A drop in blood pressure was observed in 30 patients (44.1%), with 27 patients (39.7%) experiencing a decrease of ≥30% from baseline. Bradycardia occurred in 30 patients (44.1%). The frequency of blood pressure decrease and a ≥30% drop in blood pressure compared to baseline was significantly higher in patients with early gastric cancer and gastric adenoma compared to those with superficial esophageal cancer (*p* = 0.012, *p* = 0.030). Ephedrine hydrochloride and atropine sulfate were administered to 32 (47.1%) and 16 (23.5%) patients, respectively. No treatment interruptions occurred due to patient movement. None of the patients experienced serious adverse events that were not improved with the use of ephedrine hydrochloride or atropine sulfate (Table 6).

**TABLE 6:** Safety of sedation.

		*n* = 68	Superficial esophageal cancer (*n* = 14)	Early gastric cancer and gastric adenoma (*n* = 54)	*p*‐value
Body movement to the extent that treatment is interrupted	Yes/no	0 (0%)/68 (100%)	0 (0%)/14 (100%)	0 (0%)/54 (100%)	−
Increased oxygen*	Yes/no	13 (19.1%)/55 (80.9%)	1 (7.1%)/13 (92.9%)	12 (22.2%)/42 (77.8%)	0.204
Maximum oxygen flow rate	Median (range), (L/min)	2 (2–10)	2 (2–4)	2 (2–10)	0.219
SpO2 (minimum value during treatment)	Median (range), (%)	97 (88–100)	98 (94–99)	97(88–100)	0.109
SpO2 <90% (minimum value during treatment)	Yes	1 (1.5%)	0 (0%)	1 (1.9%)	0.611
Nasal airway insertion	Yes	0 (0%)	0 (0%)	0 (0%)	−
Hypotension (<90 mmHg)	Yes	30 (44.1%)	2 (14.3%)	28 (51.9%)	0.012
Drop in blood pressure of ≥30% compared to baseline	Yes	27 (39.7%)	2 (14.3%)	25 (46.3%)	0.030
Bradycardia (pulse <50 bpm)	Yes	30 (44.1%)	5 (35.7%)	25 (46.3%)	0.481
Treatment was interrupted except for body movement	Yes	1 (1.5%)	0 (0%)	1 (1.9%)	0.611
Additional doses of haloperidol or flunitrazepam	Yes/no	0 (0.0%)/68 (100.0%)	0 (0%)	0 (0%)	−
Administration of ephedrine hydrochloride	Yes/no	32 (47.1%)/36 (52.9%)	5 (35.7%)/9 (54.3%)	27 (50.0%)/27 (50.0%)	0.344
Administration of atropine sulfate	Yes/no	16 (23.5%)/52 (76.5%)	4 (28.6%)/10 (71.4%)	12 (22.2%)/42 (77.8%)	0.620

A total of 22 non‐anesthesiologists, including 14 physicians in their 3rd year, seven in their 5^th^ year, and one in their 12^th^ year performed sedation. Among them, there was one specialist in gastrointestinal endoscopy and 21 non‐specialists. All 22 non‐anesthesiologists passed the proficiency evaluation.

**FIGURE 3:**
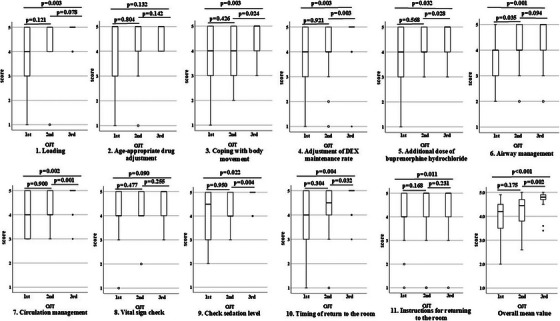
Scoring results and overall mean values for the 11 evaluation items of the first, second, and third on‐the‐job training (OJT) sessions. OJT, on‐the‐job training.

Figure 3 shows the scoring results and overall mean values for the 11 evaluation items across the first, second, and third OJT sessions. The overall mean scores for the 11 proficiency items among non‐anesthesiologists progressively improved from the first to the third OJT session.

## DISCUSSION

Sedation with DEX and MDZ, administered according to the sedation regimen, was considered feasible, with few instances of airway stenosis or body movement that interrupted the treatment despite some circulatory changes, including hypotension and bradycardia.

DEX is a selective α2 adrenergic receptor agonist that activates α2A receptors in the nucleus accumbens and spinal cord of the cerebral bridge, providing sedative, analgesic, and sympathoinhibitory effects with minimal respiratory depression.^12^

Takimoto et al. conducted a three‐arm comparison study on sedation during ESD for early gastric cancer, comparing DEX, MDZ, and propofol. They reported significantly fewer instances of body movement and a decrease in SpO_2_ that interrupted treatment in the DEX group compared to the MDZ or propofol groups. They also observed a 17% decrease in blood pressure of ≥30% from baseline, though this was not significantly different from the MDZ and propofol groups.^13^

Yoshio et al. reported that sedation using DEX and MDZ during ESD for superficial esophageal cancer was beneficial, with less body movement and respiratory depression than MDZ alone, with approximately 90% of endoscopists satisfied with the sedation.^14^ However, as patients were sedated to deep levels (Ramsay sedation score of 4–6) using a DEX loading dose after 3 mg of MDZ and 17.5 mg of pethidine, the incidence of respiratory depression was notably high, affecting 23% of patients. Additionally, 80% of patients experienced respiratory depression within 4 min of treatment initiation.

In ESD for superficial esophageal cancer, many patients were heavy alcohol consumers, which may reduce the effectiveness of sedation with benzodiazepines, leading to treatment interruptions due to body movements.^15^, ^16^ In contrast, in this study, ESD was performed with moderate sedation, targeting a RASS scale of −2 to −4. Respiratory depression or body movements that interrupted treatment were rare, and all cases were completely resected en bloc. Although hypotension and bradycardia occurred in 44.1% of patients, all patients were treated with ephedrine hydrochloride or atropine sulfate, which is comparable to that in previous reports. Thus, in this study, the sedation regimen was effective in ensuring safe sedation.

Additionally, an overall increase in the scores and mean values for the 11 proficiency items was noted after three OJT sessions for non‐anesthesiologists. This suggests that conducting three OJT sessions, rather than two, may enhance the safety and effectiveness of sedation with DEX and MDZ.

The OJT program was conducted to ensure safety when DEX was introduced for endoscopic procedures. Although the effectiveness of the OJT remains to be fully validated, it is believed that non‐anesthesiologists acquired essential knowledge about sedation through the web‐based training program. The introduction of DEX under anesthesiologists' supervision raised awareness of sedation techniques, allowing for its safe administration. The efficacy of OJT will be further evaluated as more cases are included in future studies.

This study had certain limitations. It was a single‐center, retrospective, observational study. No randomized controlled trials have compared DEX and MDZ sedation to other sedation methods. Additionally, the presence of an anesthesiologist during the procedures may have influenced the sedation outcomes. Selection bias, from the perspective of clinical practice, also cannot be ruled out. Therefore, our findings should be validated with larger sample sizes and additional clinical studies.

In Japan, unlike Europe and the United States, assigning anesthesiologists to endoscopic procedures is challenging because of the chronic shortage of anesthesiologist personnel.^3^ To minimize the risk of sedation‐related incidents by non‐anesthesiologists, we believe that conducting thorough patient risk assessments, assigning sedation personnel separate from the endoscopist, and establishing clear guidelines are important for sedation protocols.

In conclusion, a sedation regimen combining DEX and MDZ can be safely administered by non‐anesthesiologists. However, further validation studies are needed to assess its appropriateness.

## CONFLICT OF INTEREST STATEMENT

None.

## ETHICS STATEMENT

The current study adhered to the principles outlined in the Declaration of Helsinki and received approval from the Ethics Committee of the Kitasato University School of Medicine Hospital (B21‐207).

## PATIENT CONSENT STATEMENT

Informed consent (with the option to opt‐out) was obtained from all patients in compliance with the Japanese guidelines for human medical research.

## CLINICAL TRIAL REGISTRATION

N/A.

## References

[1] Cohen LB , Delegge MH , Aisenberg J *et al*. AGA Institute review of endoscopic sedation. Gastroenterology 2007; 133: 675–701. 17681185 10.1053/j.gastro.2007.06.002

[2] Metzner J , Posner KL , Domino KB . The risk and safety of anesthesia at remote locations: The US closed claims analysis. Curr Opin Anaesthesiol 2009; 22: 502–508. 19506473 10.1097/ACO.0b013e32832dba50

[3] Japanese Society of Anesthesiologists . Proposal of the Japanese Society of Anesthesiologists for the shortage of anesthesiologist manpower. Accessed: October 2, 2024 (In Japanese). https://anesth.or.jp/files/download/news/suggestion20050209_1.pdf

[4] Early DS , Lightdale JR , Vargo JJ *et al*. Guidelines for sedation and anesthesia in GI endoscopy. Gastrointest Endosc 2018; 87: 327–337. 29306520 10.1016/j.gie.2017.07.018

[5] Gotoda T , Akamatsu T , Abe S *et al*. Guidelines for sedation in gastroenterological endoscopy (second edition). Dig Endosc 2021; 33: 21–53. 33124106 10.1111/den.13882

[6] Inatomi O , Imai T , Fujimoto T *et al*. Dexmedetomidine is safe and reduces the additional dose of midazolam for sedation during endoscopic retrograde cholangiopancreatography in very elderly patients. BMC Gastroenterol 2018; 18: 166. 30400828 10.1186/s12876-018-0897-5 PMC6219039

[7] American Society of Anesthesiologists . ASA physical status classification system. Accessed: October 2, 2024. https://www.asahq.org/resources/clinical‐information/asaphysicalstatus/classification/system

[8] Charlson ME , Pompei P , Ales KL *et al*. A new method of classifying prognostic comorbidity in longitudinal studies: Development and validation. J Chronic Dis 1987; 40: 373–383. 3558716 10.1016/0021-9681(87)90171-8

[9] Japanese Gastric Cancer Association . Gastric Cancer Treatment Guideline, 6th edn, Tokyo: Kanehara, 2021.

[10] National Cancer Institute . Common terminology criteria for adverse events (CTCAE). Ver. 5.0. Accessed: October 2, 2024. https://ctep.cancer.gov/protocoldevelopment/electronic_applications/docs/ctcae_v5_quick_reference_5x7.pdf

[11] Sessler CN , Gosnell MS , Grap MJ *et al*. The Richmond Agitation‐Sedation Scale: Validity and reliability in adult intensive care unit patients. Am J Respir Crit Care Med 2002; 166: 1338–1344. 12421743 10.1164/rccm.2107138

[12] Hashiguchi K , Matsunaga H , Higuchi H *et al*. Dexmedetomidine for sedation during upper gastrointestinal endoscopy. Dig Endosc 2008; 20: 178–183.

[13] Takimoto K , Ueda T , Shimamoto F *et al*. Sedation with dexmedetomidine hydrochloride during endoscopic submucosal dissection of gastric cancer. Dig Endosc 2011; 23: 176–181. 21429025 10.1111/j.1443-1661.2010.01080.x

[14] Yoshio T , Ishiyama A , Tsuchida T *et al*. Efficacy of novel sedation using the combination of dexmedetomidine and midazolam during endoscopic submucosal dissection for esophageal squamous cell carcinoma. Esophagus 2019; 16: 285–291. 30937573 10.1007/s10388-019-00666-z

[15] Lieber CS . Alcohol and the liver: 1994 update. Gastroenterology 1994; 106: 1085–1105. 8143977 10.1016/0016-5085(94)90772-2

[16] Weathermon R , Crabb DW . Alcohol and medication interactions. Alcohol Res Health 1999; 23: 40–54. 10890797 PMC6761694

